# Multi-sensor fusion outperforms single indicators for fatigue prediction in university soccer players: a machine learning approach

**DOI:** 10.3389/fphys.2026.1775906

**Published:** 2026-02-18

**Authors:** Xuezhu Xu

**Affiliations:** School of Physical Education, Physical Education and Training, Guizhou Normal University, Guiyang, China

**Keywords:** collegiate soccer, fatigue assessment, machine learning, training load, wearable technology

## Abstract

**Background:**

Collegiate football players face unique challenges balancing academic and athletic demands, yet research on multi-sensor training load monitoring for this population remains limited.

**Objective:**

To evaluate multi-sensor wearable devices for training load monitoring and fatigue prediction in collegiate football players.

**Methods:**

Forty-eight male collegiate football players were monitored over 12 weeks using GPS devices, heart rate monitors, and subjective questionnaires. External and internal load indicators were collected during 536 training sessions and 24 matches. Fatigue status was defined using countermovement jump, heart rate variability, wellness scores, and RPE. XGBoost, random forest, and logistic regression models were developed and validated.

**Results:**

Strong correlations existed between external and internal load indicators (Player Load vs. TRIMP: r = 0.81). The XGBoost model achieved optimal performance (AUC = 0.895), significantly outperforming single-indicator models. Wellness score (18.5%), ACWR (16.2%), and morning HRV (13.8%) were the most important predictive features. Position-specific load patterns were observed, with midfielders covering greatest distances and forwards showing highest sprint outputs.

**Conclusion:**

Multi-sensor fusion combined with machine learning (XGBoost, AUC = 0.895) significantly outperforms single-indicator models for fatigue prediction in university soccer players, with wellness score, ACWR, and morning HRV identified as the most important predictive features.

## 1 Introduction

In recent years, with increasing national emphasis and sustained investment in campus football development, collegiate football in China has entered a period of rapid growth. The scale of national collegiate football leagues continues to expand, with competitive standards and public attention significantly improving. Collegiate football players represent a crucial talent pool for the sport’s development, positioned at a critical transitional stage from amateur to professional training. The quality of their development directly impacts the long-term future of Chinese football. Achieving scientific management of collegiate football training to improve training efficiency and athletic performance has become an urgent practical challenge.

Collegiate football players constitute a unique population with distinctive characteristics. Compared to professional athletes, their training duration is relatively shorter, with considerable room for development in physical fitness reserves and tactical capabilities. More notably, collegiate athletes face the challenge of balancing “dual academic and athletic responsibilities,” requiring them to manage limited time and energy between academic requirements and training demands (Lopes Dos Santos et al., 2020). Academic pressure, examination anxiety, and future career decisions may affect athletes’ psychological states, subsequently interfering with training adaptation and recovery processes. Additionally, the relatively irregular lifestyle of college students, including insufficient sleep duration, disrupted schedules, and unbalanced nutrition, may similarly impact athletic performance and physical recovery.

Scientific training monitoring holds particular significance for collegiate football players. Given that training time is constrained by course schedules, collegiate athletes typically have lower weekly training volumes than professional players, making the efficiency of each training session critically important. Scientific load monitoring can help coaches maximize training effectiveness within limited time, ensuring that training stimuli match athletes’ tolerance capacity. Simultaneously, accurate fatigue assessment enables early identification of overtraining symptoms, preventing injuries and performance decrements resulting from fatigue accumulation (Owoeye et al., 2020). Improper training load management poses dual risks: overtraining leads to persistent fatigue and increased injury risk, while undertraining fails to elicit effective training adaptations and improvements in physical capacity. Therefore, identifying the optimal load balance point for each individual represents the core objective of training management.

However, current collegiate football training commonly suffers from outdated monitoring methods and insufficient scientific sophistication. Many collegiate football teams lack professional strength and conditioning coaches and sports science personnel support. Coaches often arrange training based on personal experience, lacking objective quantitative monitoring tools and data-driven decision support. Training load assessment largely relies on subjective perception, making it difficult to accurately gauge athletes’ true conditions. Fatigue issues are often only identified when symptoms become obvious, missing optimal windows for early intervention.

The rapid development of wearable technology provides new possibilities for addressing the above challenges (Seshadri et al., 2021). GPS positioning devices, heart rate monitoring systems, and inertial measurement units have become increasingly mature, with device sizes continuously shrinking, prices declining, and operation becoming simpler. These devices can collect real-time multidimensional data including running distance, speed, heart rate, and acceleration, providing technical support for objective quantification of training load. Integration analysis of multi-sensor data, combined with artificial intelligence technologies such as machine learning, holds promise for achieving accurate prediction and early warning of athlete fatigue status. Recent studies have demonstrated the effectiveness of machine learning approaches in this domain. Rossi et al. (2022a) developed an XGBoost-based framework using GPS and RPE data to forecast wellness status in elite soccer players, achieving 87% prediction accuracy with SHAP analysis revealing chronic workload as the most important predictor. Piłka et al. (2023) applied XGBoost to GPS-derived features from Catapult wearables, achieving 90% accuracy in predicting fatigue-related injury risk, with acceleration/deceleration patterns and high-speed running distance identified as key predictive features. Similarly, Simonelli et al. (2025a) employed machine learning and mediation analysis to explore determinants of subjective recovery in 101 professional soccer players, demonstrating that fatigue and muscle soreness mediate the relationship between training load and recovery status. However, existing research primarily focuses on professional athletes (Bourdon et al., 2017; Thorpe et al., 2017). Collegiate athletes differ significantly from professional athletes in physiological characteristics, training conditions, and lifestyle (Lopes Dos Santos et al., 2020), yet research on multi-sensor fusion monitoring and fatigue prediction for this population remains virtually nonexistent (Dudley et al., 2023).

Based on this background, this study aimed to explore the application effects of multi-sensor wearable devices for training load monitoring and fatigue assessment in collegiate football players. Through 12-week longitudinal tracking of collegiate football players, collecting GPS, heart rate, and subjective questionnaire multidimensional data, this study analyzed relationship characteristics between external and internal loads, established machine learning-based fatigue prediction models, and explored load specificity among different playing positions. Specifically, this study addressed three research questions:

1. Can multi-sensor indicators effectively reflect training load characteristics? We hypothesized significant correlations between external and internal load indicators (H1).
2. Which indicator combinations yield optimal fatigue prediction performance? We hypothesized that multi-sensor fusion models would outperform single-indicator models (H2).
3. Do load-fatigue relationships exhibit position specificity? We hypothesized significant differences in load characteristics among playing positions (H3).

This study contributes to current knowledge in three ways: (1) validating the effectiveness of multi-sensor monitoring in collegiate athletes, a population underrepresented in existing literature; (2) developing and comparing machine learning models for fatigue prediction with quantified performance metrics; and (3) identifying position-specific load patterns to inform individualized training strategies.

## 2 Literature review

### 2.1 Theoretical foundation of training load monitoring

Training load is one of the core concepts in sports training science. Based on the revised conceptual framework proposed by Jeffries et al. (2022), training-related constructs can be categorized into three distinct components: external training load, internal training load, and training effects.

External Training Load refers to the objective physical work completed by athletes during training or competition, quantifiable through metrics such as distance, speed, acceleration, and power, reflecting “what was done” during training.

Internal Training Load refers to athletes’ responses to external load stimuli and can be further divided into two subcategories: (1) Objective Internal Load, which includes measurable physiological responses such as heart rate, blood lactate, and oxygen uptake; and (2) Subjective Internal Load, which encompasses psychological perceptions such as rating of perceived exertion (RPE), reflecting “how hard” the training felt.

Training Effects represent the outcomes resulting from the interaction between external and internal loads. These effects can be classified along two dimensions: temporal (acute vs. chronic) and valence (positive vs. negative). Acute negative effects include fatigue and muscle soreness, while chronic positive effects include fitness adaptations. Importantly, fatigue should be conceptualized as a training effect rather than a component of training load, as it represents the consequence of load exposure rather than the load itself (Jeffries et al., 2022). Impellizzeri et al. (2019) emphasized that the relationship between external and internal load is not simply linear. Earlier work by Impellizzeri et al. (2005) established foundational methods for physiological assessment of aerobic training in soccer. The relationship between external and internal load is not simply linear; the “coupling degree” between them is itself important information reflecting athlete status.

To achieve scientific training load management, researchers have proposed various quantification models. The Training Impulse (TRIMP) model, proposed by Banister (1991), calculates load by combining exercise duration and intensity based on heart rate data. The session-RPE (sRPE) method, proposed by Foster et al. (2001), quantifies load using the product of RPE and training duration, offering simplicity of operation and moderate to high correlation with heart rate-based TRIMP measures, though correlation strength varies considerably across training contexts. A systematic review by Haddad et al. (2017) reported correlations ranging from r = 0.50 to r = 0.90 depending on sport type and training modality, with stronger correlations typically observed during steady-state endurance activities (r = 0.70–0.85) compared to high-intensity intermittent training (r = 0.31–0.46). More recently, Foster et al. (2021) provided a comprehensive 25-year historical perspective on the development and validation of the session-RPE method. Depending on sport type and training modality, with stronger correlations typically observed during steady-state endurance activities (r = 0.70–0.85) compared to high-intensity intermittent training (r = 0.31–0.46). The Acute: Chronic Workload Ratio (ACWR) model, systematically elaborated by Gabbett (2016), evaluates the balance between short-term and long-term loads. Gabbett proposed that maintaining ACWR between 0.8–1.3 corresponds to lower injury risk, while exceeding 1.5 significantly increases risk—a theory known as the “training-injury prevention paradox.” However, the predictive validity of ACWR has been subject to recent debate. A 10-month randomized controlled trial in elite youth football found no significant reduction in injury or health problems using ACWR-guided load management compared to usual training methods (Enright et al., 2020). Systematic reviews have also indicated that ACWR’s predictive value is inconclusive and context-dependent, influenced by calculation methods (rolling average vs. exponentially weighted moving average) and sport-specific conditions (Impellizzeri et al., 2020; Griffin et al., 2020; Wang et al., 2020). While ACWR remains a useful heuristic for monitoring load progression, practitioners are advised to apply it cautiously as one component within a comprehensive monitoring framework rather than as a standalone predictor. Additionally, training monotony and training strain are important monitoring indicators; Foster’s (1998) research demonstrated strong associations between high training strain and overtraining and illness incidence.

### 2.2 Application of wearable devices in sports monitoring

Global Positioning System (GPS) technology has been applied to sports training monitoring since the early 21st century and has become the mainstream approach for external load assessment in team sports. Current mainstream sports GPS devices have sampling frequencies of 10–18 Hz, capable of accurately monitoring high-intensity activities such as short sprints and rapid directional changes. In football, commonly monitored GPS indicators include total distance, high-speed running distance, sprint distance, acceleration/deceleration counts, and maximum velocity. Akenhead and Nassis (2016) noted that GPS technology has been widely adopted in high-level football training monitoring. Ravé et al. (2020) provided practical guidance on using GPS data to monitor training load in elite soccer settings. The inter-device reliability of GPS systems has been validated across multiple days of team sport movement (Crang et al., 2022). However, GPS signals are affected by environmental factors and cannot function properly in indoor or heavily obstructed environments.

Heart rate monitoring is the most commonly used physiological indicator for assessing exercise internal load. Modern devices primarily employ two technical approaches: chest-strap electrocardiographic monitoring and photoplethysmography (PPG). Beyond real-time heart rate, heart rate recovery (HRR) and heart rate variability (HRV) also hold important value in training monitoring. HRV reflects autonomic nervous system regulation of cardiac rhythm, with RMSSD primarily reflecting parasympathetic activity and serving as a sensitive indicator for monitoring recovery status and fatigue accumulation. Plews et al. (2013) demonstrated that sustained decreases in morning HRV can serve as early warning signals for overtraining. Lundstrom et al. (2023) recently reviewed current practices and applications of HRV monitoring in endurance athletes.

Inertial Measurement Units (IMUs) integrate accelerometers, gyroscopes, and magnetometers, capable of measuring athletes’ motion information in three-dimensional space. Gómez-Carmona et al. (2020) conducted a systematic review confirming accelerometry as a valid method for external workload monitoring in invasion team sports. The “Player Load” metric calculated from IMU data has gained widespread application, capturing jumps, contacts, directional changes, and other activities that do not produce displacement but consume energy. Current mainstream monitoring devices integrate GPS and IMU technologies, achieving complementary advantages.

Multi-sensor fusion represents an important trend in sports monitoring. Single sensors cannot comprehensively reflect the multidimensional characteristics of training load, while multi-source data integration can provide richer and more accurate assessment information. Bourdon et al. (2017), in an international consensus statement, indicated that comprehensive use of multiple monitoring tools is the recommended strategy for effective athlete load management. In recent years, the introduction of machine learning techniques has provided more powerful tools for multi-sensor data fusion, capable of automatically extracting feature combinations related to target variables from multidimensional data.

### 2.3 Research on fatigue assessment in football players

Fatigue is conceptualized as an acute negative training effect, representing temporary decreases in work capacity resulting from the interaction between external load exposure and internal physiological responses (Jeffries et al., 2022). It is important to distinguish fatigue from effort: while effort (measured via RPE) reflects the subjective internal load experienced during training, fatigue represents the subsequent outcome or consequence of that training stimulus. Based on mechanism, fatigue can be categorized into peripheral and central fatigue; from a temporal dimension, it can be classified as acute or cumulative fatigue. If cumulative fatigue is not addressed in time, it may develop into functional overreaching or even overtraining syndrome. Bestwick-Stevenson et al. (2022) provided a comprehensive narrative review of fatigue and recovery assessment methods in sport.

Football player fatigue assessment can be conducted from multiple dimensions. Regarding physiological indicators, sustained decreases in morning HRV are closely associated with training load accumulation and fatigue status, while abnormal increases in resting heart rate also serve as warning signals. For neuromuscular function, the countermovement jump (CMJ) test has been widely adopted due to its simplicity and high sensitivity; Claudino et al. (2017) meta-analysis demonstrated CMJ’s good sensitivity for detecting football player fatigue. For subjective perception, the athlete wellness questionnaire proposed by Hooper and Mackinnon (1995) encompasses dimensions including fatigue sensation, sleep quality, muscle soreness, stress level, and mood. Duignan et al. (2020) conducted a systematic review examining single-item self-report measures of team-sport athlete wellbeing and their relationship with training load. Saw et al.'s (2016) systematic review found that subjective self-report indicators often show better sensitivity to training load changes than objective physiological indicators. Biochemical markers such as creatine kinase, cortisol, and testosterone can provide objective evidence but involve high testing costs and cannot be implemented frequently.

Regarding load-fatigue-injury relationships, Gabbett’s (2016) “training-injury prevention paradox” proposes that the key issue lies not in absolute load levels but in load change patterns. Moderate high-load training can enhance load tolerance capacity, while both rapid load increases and prolonged low-load training increase injury risk. Beato et al. (2021) provided scientific rationale and methodological recommendations for implementing strength training strategies for injury prevention in soccer. Fatigue plays a mediating role between load and injury; Thorpe et al.'s (2017) research demonstrated that comprehensive monitoring of fatigue markers can identify high-injury-risk states.

### 2.4 Characteristics of collegiate athletes

Collegiate athletes (aged 18–22) have essentially completed physiological development but typically have lower competitive ability and training experience than professional athletes. This population is at a critical transitional stage from amateur to professional training, facing unique multiple pressures. Beyond training and competition pressures, they must also cope with academic requirements, examination stress, and future career choices. This “dual academic-athletic responsibility” creates heavier psychological burdens for collegiate athletes, potentially interfering with training adaptation and recovery processes through effects on sleep quality and neuroendocrine function. Additionally, the relatively irregular lifestyle of college students presents widespread issues of insufficient sleep duration and variable schedules.

Regarding training conditions, collegiate athlete training is typically scheduled during extracurricular time and constrained by course schedules, challenging training continuity and systematicity. Collegiate football coaching teams are generally smaller and less specialized than professional clubs, with many universities lacking dedicated strength coaches and sports science personnel. Resources for facilities, medical rehabilitation, and nutritional support are relatively limited. These constraints highlight the necessity of establishing simple, practical, and cost-effective load monitoring systems.

Current research on training monitoring for collegiate football players remains relatively scarce. Existing studies have found that collegiate athletes’ load levels are lower than professional players, but the gap between match and training loads is relatively larger, suggesting insufficient training-competition load matching. For fatigue monitoring, most studies directly apply professional athlete criteria, whose applicability to collegiate populations has not been adequately verified. Dudley et al. (2023) conducted a systematic review and best-evidence synthesis examining methods of monitoring internal and external loads and their relationships with physical qualities, injury, or illness in adolescent athletes. Multi-sensor fusion load monitoring and fatigue assessment research for collegiate athletes presents an obvious gap requiring targeted investigation.

### 2.5 Application of machine learning in sports science

Machine learning, as an important branch of artificial intelligence, has been increasingly applied in sports science in recent years. Its core advantage lies in automatically learning patterns and rules from complex multidimensional data to construct predictive models. Compared to traditional statistical methods, machine learning algorithms can automatically capture nonlinear relationships and higher-order interaction effects, offering stronger modeling capabilities for complex systems.

Commonly used machine learning algorithms include logistic regression, decision trees, random forests, support vector machines, and gradient boosting methods (e.g., XGBoost). Among these, random forests and XGBoost, as ensemble learning methods that improve predictive performance by combining multiple weak learners, are frequently applied in sports science research and can output feature importance rankings to enhance model interpretability. Claudino et al. (2019) systematic review summarized the current application status of machine learning in sports injury prediction. Van Eetvelde et al. (2021) conducted a systematic review specifically examining machine learning methods in sport injury prediction and prevention. Claudino et al. (2019) systematic review summarized the current application status of machine learning in sports injury prediction, noting the method’s advantages in integrating multi-source data and handling nonlinear relationships.

For fatigue assessment, machine learning can be used to establish fatigue status classification or prediction models, with input features potentially including external load, internal load, physiological monitoring, and subjective assessment multidimensional data. Compared to traditional single-threshold methods, machine learning models can comprehensively consider joint effects of multiple indicators, potentially achieving more accurate fatigue identification. However, data quality and sample size are key factors constraining model performance. Sports science research samples are typically small, requiring attention to preventing overfitting. Additionally, the “black box” nature of models may limit in-depth understanding of prediction mechanisms, necessitating balance between predictive performance and interpretability in practical applications.

Recent large-scale studies have further advanced machine learning applications for fatigue prediction in professional soccer. Simonelli et al. (2025b) analyzed 30,211 observations from six Italian professional teams and found that subjective wellness indicators—particularly stress and mood—were more influential predictors of match-day fatigue than training load metrics alone. Their findings suggest that daily individual fluctuations in wellness status may provide more actionable information for fatigue prediction than ratio-based workload metrics, highlighting the value of integrating subjective assessments into multi-sensor monitoring frameworks.

## 3 Materials and methods

### 3.1 Study design

This study employed a prospective longitudinal observational design to explore the application effects of multi-sensor wearable devices for training load monitoring and fatigue assessment in collegiate football players. The study period was set at 12 weeks, covering a complete training cycle to ensure adequate collection of load data across different training phases and observation of dynamic changes in fatigue status. To ensure data collection continuity and completeness, the study period was selected to avoid midterm and final examination weeks or other special periods that might significantly disrupt training schedules. The 12-week monitoring period corresponded to the in-season competitive phase of the university football calendar, during which the team participated in regular league competitions. Based on the training periodization employed by the coaching staff, the study period encompassed three distinct phases: a progressive loading phase (Weeks 1–4), a load maintenance phase (Weeks 5–10), and a pre-competition taper phase (Weeks 11–12). A total of 24 matches were completed during this period, with varying match density across different weeks depending on the league schedule.

For data collection frequency, this study adopted a multi-level, multi-timepoint monitoring strategy: real-time load monitoring during each training session and match, daily morning heart rate variability and subjective status assessment, and weekly neuromuscular function testing at fixed times. This multi-frequency data collection protocol can comprehensively capture athletes’ training load accumulation processes and fatigue status evolution patterns, providing adequate data support for subsequent fatigue prediction model development.

### 3.2 Participants

#### 3.2.1 Inclusion and exclusion criteria

Inclusion criteria for this study were: (1) age between 18 and 22 years; (2) full-time undergraduate or graduate student status; (3) officially registered member of a collegiate football team; (4) minimum of 2 years systematic football training; (5) participation in training at least 3 times per week during the study period; (6) no major injury history within the past 6 months, capable of normal participation in training and competition.

Exclusion criteria included: (1) acute injury during the study period causing training cessation exceeding 2 weeks; (2) inability to continuously participate in data collection due to personal reasons, resulting in data missing rate exceeding 20%; (3) withdrawal from the team during the study period or inability to continue participation for other reasons.

#### 3.2.2 Sample size estimation

Sample size was estimated using G*Power 3.1 software. Based on effect sizes reported in prior literature (Cohen’s d = 0.5), with two-sided test significance level α = 0.05 and statistical power (1-β) = 0.80, the minimum required sample size was calculated as 34 participants. Considering potential sample attrition in longitudinal research, the target recruitment sample size was adjusted upward by approximately 20% to 45–50 participants, ensuring at least 40 valid samples at study completion.

#### 3.2.3 Participant sources

Participants for this study were primarily recruited through the following channels: first, recruitment through the university’s high-level football team, which has a stable training schedule and high competitive level; second, recruitment among sports college football-specialized students, who possess good football technical foundation and high research compliance. If single-institution sample size proved insufficient, multi-center recruitment in collaboration with neighboring university football teams could be considered to expand sample sources and enhance external validity of research conclusions. The final sample comprised 48 male collegiate football players (4 withdrew: 2 due to injury, 1 team withdrawal, 1 data incompleteness). Mean age was (20.3 ± 1.2) years, height (176.5 ± 5.8) cm, body mass (68.7 ± 6.4) kg, and body fat (12.4 ± 2.8)%. Football training experience was (7.2 ± 2.5) years. Position distribution: defenders (n = 15, 31.3%), midfielders (n = 18, 37.5%), forwards (n = 12, 25.0%), and goalkeepers (n = 3, 6.2%). Baseline physiological measures included maximum heart rate (196.4 ± 7.3) bpm, resting heart rate (58.2 ± 6.1) bpm, and CMJ height (38.6 ± 4.2) cm. Complete participant characteristics are presented in Table 1.

**TABLE 1:** Participant characteristics (n = 48).

Variable	Mean ± SD/n (%)
Age (years)	20.3 ± 1.2
Height (cm)	176.5 ± 5.8
Body mass (kg)	68.7 ± 6.4
Body fat (%)	12.4 ± 2.8
BMI (kg/m^2^)	22.0 ± 1.5
Training experience (years)	7.2 ± 2.5
Maximum heart rate (bpm)	196.4 ± 7.3
Resting heart rate (bpm)	58.2 ± 6.1
Baseline CMJ height (cm)	38.6 ± 4.2
Playing position	
Goalkeeper	3 (6.2%)
Defender	15 (31.3%)
Midfielder	18 (37.5%)
Forward	12 (25.0%)

#### 3.2.4 Ethics statement

Ethical approval was obtained from the Institutional Review Board (Ethics approval number: IRB-GZNU-2024-156) prior to study implementation. During participant recruitment, researchers provided each potential participant with detailed information about study objectives, procedures, expected benefits, and potential risks, ensuring voluntary participation decisions based on full informed consent. All participants signed written informed consent forms before formal enrollment. Participants retained the right to withdraw from the study at any time without reason, with withdrawal decisions having no impact on their team status or other rights.

Regarding data management, all information involving participant personal identity was processed using coding, with original data and identity cross-reference tables stored separately and accessible only to core research team members. Research data were used exclusively for statistical analysis within this study and not for any other purposes. This study was conducted in full compliance with the ethical principles for medical research involving human subjects as outlined in the Declaration of Helsinki.

### 3.3 Instruments

**FIGURE 1:**
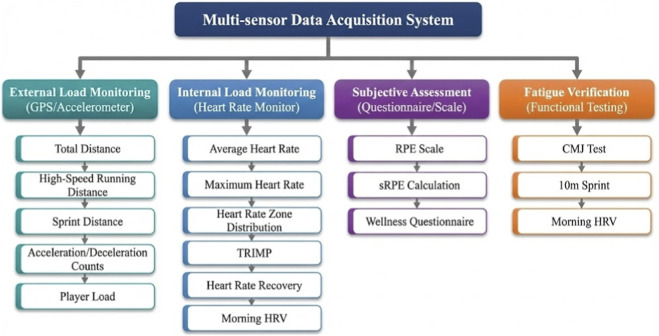
Multi-sensor data collection system framework.

This study employed a multi-sensor wearable device system for data collection, with monitoring indicators encompassing four dimensions: external load, internal load, subjective assessment, and fatigue verification. The framework of the multi-sensor data collection system is shown in Figure 1.

External load data were collected using integrated wearable devices with built-in GPS and tri-axial accelerometers. The device selected was the Catapult OptimEye S5 sports performance monitoring system (Catapult Sports, Australia), with GPS sampling frequency of 10 Hz and accelerometer sampling frequency of 100 Hz, capable of meeting monitoring requirements for high-intensity intermittent activities in football. Although this device has been superseded by the Vector series since 2019, the OptimEye S5 remains one of the most extensively validated systems in sports science literature, with over 100 peer-reviewed validation studies (Catapult Sports, n.d.). Its continued use in this study was based on equipment availability at the participating institution and the extensive body of comparable research data, facilitating cross-study comparisons. Devices were worn in vest form on athletes’ upper back between the shoulder blades, without affecting normal training and competition movements.

Internal load data were collected using chest-strap heart rate monitoring devices. The device selected was the Polar H10 heart rate sensor (Polar Electro, Finland), paired with the Polar Team Pro system for multi-person synchronized monitoring and data transmission. Heart rate sampling frequency was 1 Hz, with simultaneous recording of raw R-R interval data for subsequent heart rate variability analysis.

### 3.4 Procedures

**FIGURE 2:**
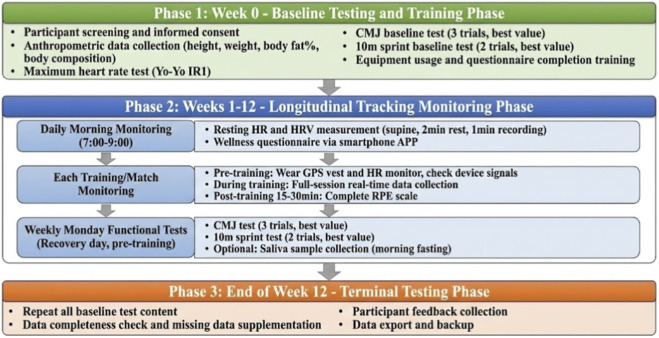
Study procedure flowchart.

Data collection procedures for this study were divided into three phases: baseline testing and training phase (Week 0), longitudinal tracking monitoring phase (Weeks 1–12), and terminal testing phase (end of Week 12). The complete study procedure is shown in Figure 2.

As shown in Figure 2, during the baseline testing and training phase, all potential participants first underwent inclusion/exclusion criteria screening, with eligible individuals signing informed consent before formal enrollment. Anthropometric data collection including height, weight, body fat percentage, and body composition analysis was then conducted. To determine individual maximum heart rate, the Yo-Yo Intermittent Recovery Test Level 1 (Yo-Yo IR1) was administered with continuous heart rate monitoring, using the highest heart rate achieved as individual maximum heart rate. Additionally, CMJ and 10-meter sprint baseline testing established individual initial reference values. Training content included proper device wearing methods, RPE scale completion protocols, Wellness questionnaire reporting procedures, and standardized operating procedures for morning HRV measurement.

The longitudinal tracking monitoring phase lasted 12 weeks, comprising three levels of data collection tasks. Daily morning monitoring required participants to complete resting heart rate and HRV measurements during a fixed time window (7:00–9:00) after waking and before breakfast, measured in supine position after 2 min of rest with 1 min of data collection. Following HRV measurement, participants completed the Wellness questionnaire via smartphone APP. During each training session or match, participants wore GPS vests and heart rate monitors for full-session monitoring, completing RPE scales within 15–30 min post-session. Mondays were designated as recovery days, with CMJ and 10-meter sprint testing conducted before that day’s training to monitor neuromuscular function status.

The terminal testing phase was scheduled at the end of Week 12, with main tasks including repeating all baseline test content, checking data completeness over the 12-week period, supplementary collection for missing data as necessary, and collecting participant feedback on the research process. Upon completion of all data collection, data export, verification, and backup work prepared for subsequent data analysis.

### 3.5 Variables

#### 3.5.1 External load indicators

External load monitoring indicators included the following aspects:

1. Distance metrics: Total Distance (m), reflecting overall activity volume during training or competition; High-Speed Running Distance (m), defined as cumulative distance at velocities exceeding 18 km/h; Sprint Distance (m), defined as cumulative distance at velocities exceeding 21 km/h (Akenhead and Nassis, 2016; Ravé et al., 2020); Relative Distance (m/min), representing distance covered per unit time, enabling standardized comparison across training sessions of different durations.
2. Velocity metrics: Peak Speed (km/h), recording maximum instantaneous velocity during monitoring; Average Speed (km/h), reflecting overall activity intensity level.
3. Acceleration/deceleration metrics: High-intensity acceleration counts, defined as cumulative count of acceleration events exceeding 2.5 m/s^2^; High-intensity deceleration counts, defined as cumulative count of deceleration events below −2.5 m/s^2^; Total acceleration/deceleration counts, reflecting overall load from velocity change activities.
4. Composite load indicator: Player Load (AU), calculated from tri-axial accelerometer data using a specific algorithm, comprehensively reflecting athletes’ activity intensity in three-dimensional space (Gómez-Carmona et al., 2020).

#### 3.5.2 Internal load indicators

##### 3.5.2.1 Objective internal load

1. Real-time training indicators: Mean heart rate (HRmean, bpm), reflecting overall cardiovascular stress during training or competition; Maximum heart rate (HRmax, bpm), recording highest heart rate during monitoring; Percentage heart rate reserve (%HRR), calculated based on individual maximum and resting heart rates, reflecting relative exercise intensity; Heart rate zone time distribution, dividing heart rate into 5 intensity zones (Zone 1–5) and recording cumulative time and proportion in each zone; Training Impulse (TRIMP, AU), calculated using Banister’s heart rate-weighted training load method, comprehensively considering exercise duration and relative intensity.
2. Recovery period indicators: Heart Rate Recovery (HRR), recording heart rate decline magnitude at 1 min (HRR-1min) and 2 min (HRR-2min) post-exercise, reflecting autonomic nervous function and cardiovascular recovery capacity.
3. Morning monitoring indicators: Resting Heart Rate (bpm), measured each morning in fasting state.

##### 3.5.2.2 Subjective Internal Load

Post-training assessment employed the Borg CR-10 Rating of Perceived Exertion (RPE) scale, using a 0–10 scoring standard where 0 represents “no exertion at all” and 10 represents “maximal exertion.” To ensure assessment accuracy, RPE completion was uniformly scheduled 15–30 min after training or competition conclusion, when athletes had completed initial recovery but could still accurately recall exertion sensations during training. Based on RPE data, session-RPE (sRPE) was calculated as: sRPE = RPE score × training duration (minutes), in arbitrary units (AU). Subjective assessment indicator collection was conducted at two timepoints: immediately post-training and daily morning assessment.

#### 3.5.3 Training effects assessment

1. Countermovement Jump (CMJ) test: Conducted using a portable photoelectric jump testing mat (OptoJump, Italy) or force platform (Claudino et al., 2017). During testing, participants stood in the center of the testing mat with hands on hips, rapidly squatted and immediately jumped upward, with legs extended during takeoff and landing. Each test included 3 valid jumps with 30-s rest intervals, recording best performance. Collected indicators included jump height (cm) and peak power (W); magnitude of jump height decline serves as an important reference for determining neuromuscular fatigue.
2. 10-meter sprint test: Conducted using an electronic timing light gate system. Participants adopted a standing start position 0.5 m behind the start line, sprinting at full effort through the 10-meter finish line upon signal. Each test included 2 trials with 2-min rest intervals, recording best time. Extended sprint time can reflect neuromuscular system fatigue accumulation.
3. Morning Heart Rate Variability (HRV): Heart Rate Variability (HRV) was measured each morning during a fixed time window (7:00–9:00) after waking and before breakfast, in supine position after 2 min of rest with 1 min of data collection. The time-domain indicator RMSSD (root mean square of successive R-R interval differences) and its natural logarithm transformation LnRMSSD were used as primary analysis indicators (Plews et al., 2013), reflecting autonomic nervous system functional status and body recovery level. Changes in morning HRV serve as a sensitive marker of training effects, particularly accumulated fatigue and recovery status.
4. Wellness Questionnaire: Daily morning assessment employed the Athlete Wellness Questionnaire, containing five dimensions: fatigue sensation, sleep quality, muscle soreness, stress level, and mood state (Perri et al., 2021). Each dimension used a one to five Likert scale, with 1 indicating worst status and 5 indicating best status. Total score ranged from 5–25, with higher scores indicating better overall wellness status. To facilitate data collection and improve compliance, the Wellness questionnaire was completed via smartphone application (APP), with athletes completing submissions each morning (7:00–9:00 time window). Wellness scores reflect acute training effects including fatigue and recovery status, rather than training load *per se*.
5. Optional biochemical indicators: When conditions permit, saliva samples may be collected on Monday mornings to test salivary cortisol (μg/dL) and salivary testosterone (pg/mL) levels. Saliva collection requires fasting state, avoiding eating, drinking, and brushing teeth within 30 min before collection. Cortisol/testosterone ratio can serve as an auxiliary reference indicator for assessing body stress-recovery balance status.

#### 3.5.4 Derived load indicators

1. Acute:Chronic Workload Ratio (ACWR): This indicator evaluates the balance between short-term and long-term loads, calculated as: ACWR = Acute Load (current week load)/Chronic Load (rolling 4-week average). This study employed Exponentially Weighted Moving Average (EWMA) to calculate ACWR (Gabbett, 2016; Williams et al., 2017).
2. Training Monotony: Reflects within-week training load variability, calculated as: Training Monotony = Weekly mean daily load/Weekly load standard deviation (Foster, 1998).
3. Training Strain: Comprehensively reflects the influence of weekly total load and load distribution pattern, calculated as: Training Strain = Weekly total load × Training monotony (Foster, 1998).

#### 3.5.5 Operational definition of fatigue status

To establish the fatigue prediction model, clear operational definition of athlete fatigue status was required. This study comprehensively integrated multiple fatigue markers, employing a multi-indicator joint determination method. Specific determination criteria are shown in Table 2.

**TABLE 2:** Fatigue status determination criteria.

No.	Determination indicator	Fatigue threshold	Reference baseline
1	CMJ jump height change	Decrease >10%	Individual 7-day rolling average
2	Morning HRV change (LnRMSSD)	Decrease >0.5	Individual 7-day rolling average
3	Wellness questionnaire total score	<15 points	Maximum 25 points
4	RPE score	≥8 for 2 consecutive days	10-point scale

When athletes simultaneously met 2 or more of the above 4 criteria on any monitoring day, they were classified as Fatigued; meeting fewer than 2 criteria was classified as Non-fatigued.

### 3.6 Data analysis

#### 3.6.1 Data preprocessing

Data preprocessing is a critical step for ensuring reliability of subsequent analyses. First, raw data from various devices and questionnaires were exported and integrated by participant code and collection date to establish a unified database. GPS and heart rate device data were exported in CSV format through respective software, with questionnaire data directly exported from the APP backend.

Outlier detection employed the 3-standard-deviation principle, flagging data points deviating more than 3 standard deviations from the mean as potential outliers. For identified outliers, original records were first verified to confirm whether they resulted from device malfunction or recording errors; confirmed outliers were removed or replaced.

Missing value handling adopted different strategies based on missing rate: variables with missing rate below 5% were processed using mean imputation; variables with missing rate between 5% and 20% were processed using Multiple Imputation, constructing multiple imputed datasets and combining analysis results to reduce imputation bias; participant data with missing rate exceeding 20% were excluded according to exclusion criteria.

To facilitate comparison across indicators with different measurement scales and subsequent machine learning modeling, all continuous variables underwent Z-score standardization: Z = (X - μ)/σ, where X is the raw value, μ is the mean, and σ is the standard deviation.

#### 3.6.2 Statistical analysis methods

Descriptive statistics were expressed as mean ± standard deviation or median (interquartile range), selected based on Shapiro-Wilk normality test results. Correlations between external and internal load indicators were analyzed using Pearson or Spearman correlation analysis. Differences between fatigued and non-fatigued groups were compared using independent samples t-tests or Mann-Whitney U tests; load differences among playing positions were compared using one-way ANOVA.

Fatigue prediction model construction employed machine learning methods, establishing logistic regression, random forest, and XGBoost models, with model performance evaluated through 10-fold cross-validation. XGBoost hyperparameters were tuned using grid search with 5-fold cross-validation on the training set. Final parameters: learning rate = 0.1, max depth = 6, n_estimators = 100, subsample = 0.8. Random forest: n_estimators = 500, max_features = ‘sqrt’. Logistic regression used L2 regularization with C = 1.0. To address potential data leakage from repeated measures within individuals, we additionally performed leave-one-subject-out cross-validation (LOSO-CV), where all data from each participant were held out in turn for testing. This approach provides a more conservative and realistic estimate of model generalizability to new individuals. Model input variables included external load, internal load, derived load indicators, and subjective assessment indicators; output variable was binary fatigue status. Model evaluation metrics included accuracy, sensitivity, specificity, F1 score, and area under the ROC curve (AUC). Single-indicator prediction models (sRPE, ACWR) were also constructed for performance comparison with multi-sensor fusion models to verify the superiority of the multi-sensor fusion approach.

To establish a performance baseline and verify that the models possessed genuine predictive capability beyond chance, a dummy classifier was included that predicted class labels based on the class distribution in the training data (i.e., stratified random prediction). This baseline represents the expected performance of a model with no actual predictive capability (AUC = 0.50), against which the trained models were compared (Rossi et al., 2022b). Additionally, to enhance model interpretability beyond standard feature importance rankings, SHapley Additive exPlanations (SHAP) analysis was conducted on the optimal model. SHAP values quantify the marginal contribution of each feature to individual predictions based on game theory, providing both global feature importance and local explanations of the model’s decision-making process (Rossi et al., 2022a).

#### 3.6.3 Statistical software and significance level

Data processing and statistical analysis employed the following software tools: SPSS 27.0 (IBM Corporation, USA) for descriptive statistics and traditional statistical tests; R 4.3.0 (R Foundation for Statistical Computing, Austria) for linear mixed model analysis and data visualization; Python 3.10 with scikit-learn, pandas, and numpy libraries for machine learning model construction and validation.

Unless otherwise specified, all statistical tests were two-sided with significance level α = 0.05, with p < 0.05 considered statistically significant. For multiple comparisons, Bonferroni correction was applied to control overall Type I error rate. Effect sizes were reported using Cohen’s d (between-group comparisons) and correlation coefficient r (correlation analyses) to facilitate evaluation of practical significance of statistical results.

## 4 Results

### 4.1 Participant characteristics

This study initially recruited 52 participants, with 4 withdrawing during the study period: 2 due to acute injuries causing training cessation exceeding 2 weeks, 1 due to personal reasons leading to team withdrawal, and 1 due to data missing rate exceeding 20%. The final valid sample for statistical analysis comprised 48 collegiate football players, yielding a sample retention rate of 92.3%.

During the 12-week data collection period, 536 training sessions and 24 matches were recorded. Each participant attended an average of (11.2 ± 1.8) training sessions per week, with data collection completeness rate of 94.6%, indicating high participant compliance and overall good data quality.

### 4.2 Training load characteristics

Descriptive statistics of load indicators for training sessions and matches during the 12-week study period are shown in Table 3.

**TABLE 3:** Descriptive statistics of training load indicators.

Category	Indicator	Training (n = 536)	Match (n = 24)	t/Z	p
External	Total distance (m)	5,765 ± 1,124	9,847 ± 1,356	−14.52	<0.001
	High-speed distance (m)	412 ± 156	876 ± 203	−11.38	<0.001
	Sprint distance (m)	98 ± 45	245 ± 78	−9.67	<0.001
	Peak speed (km/h)	26.3 ± 2.4	29.1 ± 2.1	−5.82	<0.001
	Player load (AU)	486 ± 112	892 ± 145	−13.76	<0.001
	High-intensity accel	42 ± 15	78 ± 22	−8.45	<0.001
	High-intensity decel	38 ± 14	71 ± 19	−8.12	<0.001
Internal	Mean HR (bpm)	142 ± 12	161 ± 10	−8.24	<0.001
	Max HR (bpm)	182 ± 9	193 ± 6	−6.53	<0.001
	TRIMP (AU)	186 ± 52	312 ± 68	−9.34	<0.001
	sRPE (AU)	378 ± 124	612 ± 156	−7.89	<0.001

As shown in Table 3, match loads were significantly higher than training session loads across all indicators (p < 0.001). For external load, match total distance (9,847 ± 1,356 m) was approximately 1.7 times that of training sessions (5,765 ± 1,124 m); high-speed running distance and sprint distance during matches reached (876 ± 203) m and (245 ± 78) m respectively, significantly exceeding training session levels. Match maximum velocity (29.1 ± 2.1 km/h) was approximately 10.6% higher than training sessions (26.3 ± 2.4 km/h), indicating higher velocity demands during competition. Match Player Load reached (892 ± 145) AU, approximately 1.8 times that of training sessions, reflecting substantially higher overall activity intensity during competition.

For internal load, match mean heart rate (161 ± 10 bpm) and maximum heart rate (193 ± 6 bpm) were significantly higher than training sessions. Heart rate-based TRIMP during matches reached (312 ± 68) AU, 1.7 times that of training sessions. Subjective load indicator sRPE during matches was (612 ± 156) AU, similarly significantly higher than training session levels (378 ± 124 AU). These results indicate that competition physiological stress substantially exceeds that of daily training, consistent with the high-intensity intermittent characteristics of football matches.

Regarding weekly load changes, mean weekly total distance over 12 weeks showed an initial increase followed by stabilization. Weeks 1-4 represented the load progression period, with mean weekly total distance progressively increasing from 28,540 m in Week 1–35,620 m in Week 4. Weeks 5–10 represented the load maintenance period, with mean weekly total distance maintained between 34,000–36,000 m. Weeks 11–12 represented the pre-competition adjustment period, with mean weekly total distance decreasing to approximately 30,000 m. Weekly mean sRPE showed similar trends to total distance, indicating good synchronization between external and internal loads at the macroscopic level.

### 4.3 Relationships between external and internal load

**FIGURE 3:**
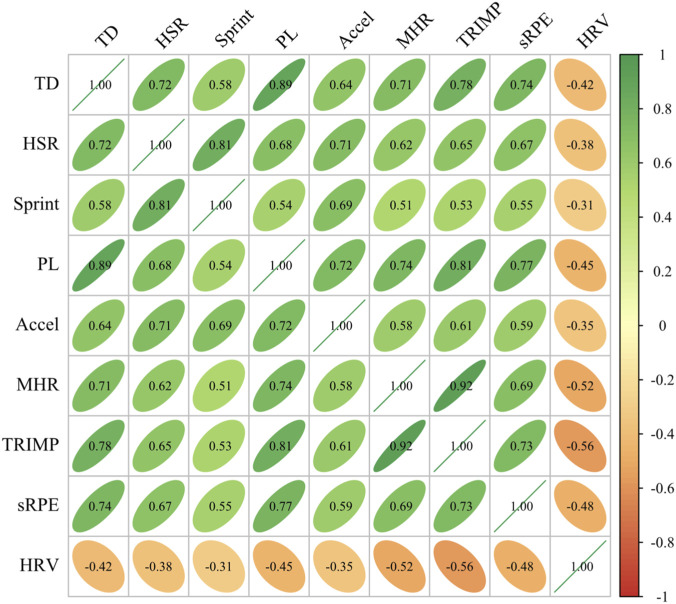
Correlation Heatmap of External and Internal Load Indicators. Note: TD, Total Distance; HSR, High-speed Running; PL, Player Load; MHR, Mean Heart Rate; HRV, Heart Rate Variability (LnRMSSD). All correlations p < 0.001.

To explore associations among various load indicators collected by multi-sensors, correlation analysis was conducted between major external and internal load indicators, with results shown in Figure 3.

As shown in Figure 3, external and internal load indicators generally showed moderate to strong positive correlations. Player Load and TRIMP demonstrated the highest correlation (r = 0.81, p < 0.0001), indicating high consistency between accelerometer-based composite load indicators and heart rate-based training impulse indicators. Total distance showed strong positive correlations with both TRIMP (r = 0.78, p < 0.0001) and sRPE (r = 0.74, p < 0.0001), validating the close relationship between external load volume and internal physiological responses.

Within external load indicators, total distance and Player Load showed the strongest correlation (r = 0.89, p < 0.0001), while high-speed running distance and sprint distance were strongly positively correlated (r = 0.81, p < 0.0001), indicating some collinearity among these indicators. Within internal load indicators, mean heart rate was highly correlated with TRIMP (r = 0.92, p < 0.0001), related to TRIMP calculation being based on heart rate data.

Notably, morning HRV (LnRMSSD) showed negative correlations with all load indicators, with the strongest negative correlation with TRIMP (r = −0.56, p < 0.0001), followed by mean heart rate (r = −0.52, p < 0.0001). This result indicates that increased training load leads to decreased next-day morning HRV, reflecting autonomic nervous system adaptive regulation in response to training stress. This finding supports the use of HRV as an effective indicator for monitoring post-training recovery status.

### 4.4 Relationships between fatigue status and load indicators

Based on the operational fatigue definition described in Section 3.6.3, among 4,032 total monitoring person-days over 12 weeks, 612 fatigue occurrences were identified, yielding a fatigue incidence rate of 15.2%. Regarding temporal distribution, fatigue primarily occurred during high-load training weeks (Weeks 4–6) and competition-intensive weeks, with fatigue incidence rate on Days 1–2 post-match (28.4%) significantly higher than regular training days (12.6%).

Comparison results of major load indicators and fatigue markers between fatigued and non-fatigued groups are shown in Table 4.

**TABLE 4:** Comparison of indicators between fatigued and non-fatigued states.

Category	Indicator	Non-fatigued	Fatigued	t/Z	p	Cohen’s d
Previous day	Total distance (m)	5,428 ± 1,089	6,834 ± 1,245	−6.72	<0.001	0.85
	sRPE (AU)	354 ± 118	498 ± 145	−6.34	<0.001	0.79
Weekly	Weekly total distance (m)	32,456 ± 4,521	38,934 ± 5,124	−7.12	<0.001	0.91
	ACWR	1.05 ± 0.22	1.38 ± 0.31	−6.89	<0.001	0.88
Fatigue	Morning HRV (LnRMSSD)	4.12 ± 0.38	3.54 ± 0.42	8.24	<0.001	1.05
Markers	Wellness total score	19.2 ± 2.4	13.6 ± 2.1	13.12	<0.001	1.67

### 4.5 Fatigue prediction model

#### 4.5.1 Feature importance analysis

**FIGURE 4:**
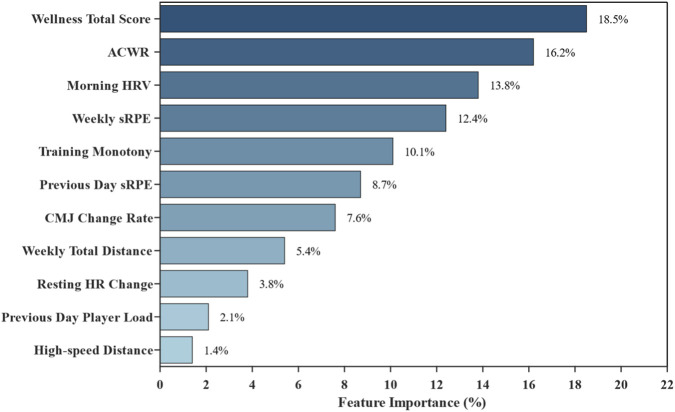
Feature importance ranking for fatigue prediction model.

Random forest algorithm was used to rank importance of all input features, with results shown in Figure 4.

As shown in Figure 4, among all input features, Wellness total score showed highest importance (18.5%), followed by ACWR (16.2%) and morning HRV (13.8%). These three indicators’ cumulative importance reached 48.5%, constituting the core feature set for fatigue prediction.

#### 4.5.2 SHAP analysis

**FIGURE 5:**
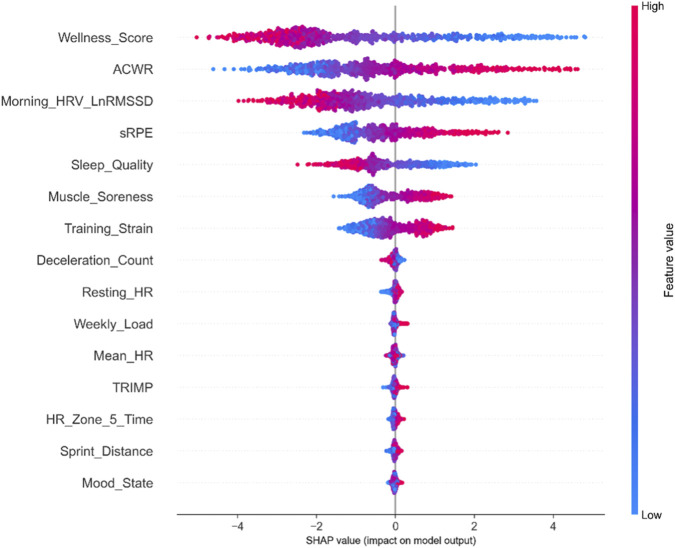
SHAP Summary Plot for XGBoost Fatigue Prediction Model. Each point represents one prediction instance. Features are ranked by mean absolute SHAP value (top = most important). Color indicates feature value (red = high, blue = low). Position on x-axis shows the impact on model output (positive = increases fatigue probability, negative = decreases fatigue probability).

To provide deeper insight into the model’s decision-making process, SHAP analysis was conducted on the optimal XGBoost model. The SHAP summary plot (Figure 5) illustrates both the importance and directional effects of each feature on fatigue prediction.

SHAP analysis revealed that Wellness score had the highest mean absolute SHAP value (0.42), with higher Wellness scores consistently associated with negative SHAP values (decreased fatigue probability). ACWR showed the second highest contribution (mean |SHAP| = 0.38), with elevated ACWR values (>1.3) associated with positive SHAP values (increased fatigue probability). Morning HRV (LnRMSSD) demonstrated the third highest contribution (mean |SHAP| = 0.31), with lower HRV values associated with increased fatigue probability.

These findings align with the random forest feature importance rankings and provide mechanistic insight: the model predicts elevated fatigue risk when athletes exhibit declining subjective wellness, rapid increases in training load relative to their chronic baseline, and reduced parasympathetic activity as indicated by lower morning HRV.

#### 4.5.3 Model performance comparison

Fatigue prediction models were constructed using logistic regression, random forest, and XGBoost machine learning algorithms, with performance compared against both a dummy classifier baseline and single-indicator prediction models. Performance metrics are shown in Table 5. The dummy classifier, which predicted fatigue based solely on class distribution (15.2% fatigue rate), achieved an AUC of 0.500, representing the expected performance of a model with no predictive capability. All trained models significantly outperformed this baseline (all p < 0.001), confirming genuine predictive validity.

**TABLE 5:** Prediction model performance comparison.

Model type	Accuracy	Sensitivity	Specificity	F1 score	AUC (95% CI)
Baseline model					
Dummy classifier	0.848 ± 0.015	0.152 ± 0.021	1.000 ± 0.000	0.264 ± 0.031	0.500 (0.500–0.500)
Multi-sensor fusion models					
Logistic regression	0.768 ± 0.042	0.711 ± 0.056	0.793 ± 0.048	0.698 ± 0.051	0.809 (0.773–0.845)
Random forest	0.836 ± 0.038	0.794 ± 0.052	0.856 ± 0.044	0.776 ± 0.047	0.878 (0.845–0.911)
XGBoost	0.851 ± 0.035	0.819 ± 0.048	0.862 ± 0.041	0.793 ± 0.044	0.895 (0.862–0.928)^a^
Single-indicator models					
sRPE only	0.658 ± 0.051	0.587 ± 0.063	0.694 ± 0.055	0.572 ± 0.058	0.687 (0.642–0.732)
ACWR only	0.694 ± 0.048	0.623 ± 0.059	0.728 ± 0.052	0.608 ± 0.054	0.724 (0.681–0.767)
Wellness only	0.745 ± 0.044	0.698 ± 0.055	0.768 ± 0.049	0.685 ± 0.052	0.786 (0.746–0.826)

Values represent mean ± SD from 10-fold cross-validation. The dummy classifier serves as a baseline representing random prediction based on class distribution (fatigue incidence = 15.2%). All trained models significantly outperformed the dummy classifier baseline (p < 0.001).

^a^ XGBoost additional validation: Leave-one-subject-out cross-validation yielded AUC = 0.847 (95% CI: 0.809–0.885), providing a more conservative estimate of generalizability to new individuals.

#### 4.5.4 Optimal model evaluation

**FIGURE 6:**
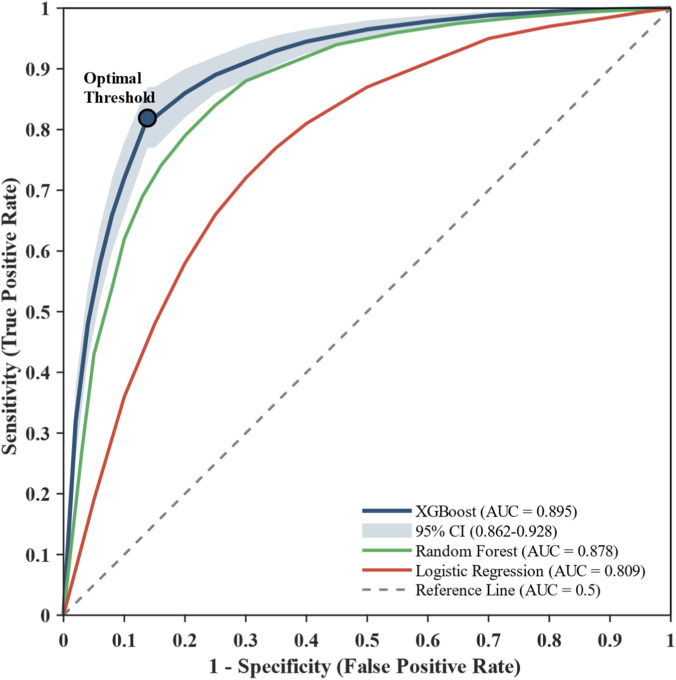
ROC Curve for XGBoost Fatigue Prediction Model. 10-fold CV: Mean AUC = 0.895 (95% CI: 0.862–0.928). At optimal threshold: Sensitivity = 0.819, Specificity = 0.862, Youden Index = 0.681. Leave-one-subject-out CV: AUC = 0.847.

Based on the comparison results, XGBoost was selected as the optimal fatigue prediction model. The ROC curve for this model is shown in Figure 6.

### 4.6 Position-specific analysis

Comparative analysis of training load characteristics was conducted among different playing positions. Due to small goalkeeper sample size (n = 3), only defenders (n = 15), midfielders (n = 18), and forwards (n = 12) were compared. One-way ANOVA results showed significant differences among the three position groups: midfielders covered greatest total distance (6,124 ± 1,089 m, F = 4.28, p = 0.019); forwards showed highest high-speed running distance (486 ± 167 m, F = 5.12, p = 0.010) and sprint distance (128 ± 52 m, F = 6.34, p = 0.004); defenders showed highest high-intensity deceleration counts (45 ± 16, F = 3.89, p = 0.027). Internal load indicators showed no significant differences among positions (p > 0.05). These results partially support Hypothesis H3.

## 5 Discussion

### 5.1 Summary of main findings

Through 12-week multi-sensor monitoring of 48 collegiate football players, this study systematically explored the application effects of wearable devices for training load monitoring and fatigue assessment. Multi-sensor wearable devices can effectively collect multidimensional training load data with 94.6% completeness rate. Significant positive correlations exist between external and internal load indicators (Player Load vs. TRIMP: r = 0.81), validating H1. The XGBoost fusion model achieved a mean AUC of 0.895 (95% CI: 0.862–0.928) under 10-fold cross-validation, significantly outperforming single-indicator models and validating H2. A more conservative leave-one-subject-out validation yielded AUC of 0.847, confirming model robustness while acknowledging the limitations of the sample size. Position-specific load characteristics were observed, partially validating H3.

#### 5.1.1 Hypothesis verification and literature comparison

Regarding our three hypotheses: H1 was fully supported, as external and internal load indicators showed strong correlations (r = 0.74–0.81). This finding is consistent with the theoretical framework proposed by Impellizzeri et al. (2019), who emphasized that external and internal loads are conceptually distinct but physiologically coupled. The strong correlation between Player Load and TRIMP (r = 0.81) also aligns with Gómez-Carmona et al. (2020), who reported similar relationships in invasion team sports.

H2 was confirmed, with the multi-sensor fusion model (AUC = 0.895) significantly outperforming single-indicator models (sRPE: 0.687; ACWR: 0.724). This improvement of 23%–30% supports the consensus recommendation by Bourdon et al. (2017) that comprehensive use of multiple monitoring tools is essential for effective athlete load management. Our results are also comparable to recent machine learning studies: Rossi et al. (2022b) achieved 87% accuracy using XGBoost for wellness prediction in elite soccer players, and Piłka et al. (2023) reported 90% accuracy for injury risk prediction using GPS-derived features.

H3 was partially supported: significant position differences were found for external load indicators (total distance, high-speed running, sprint distance, and decelerations), but not for internal load indicators. This pattern is consistent with Akenhead and Nassis (2016), who reported that positional differences are more pronounced in external rather than internal metrics. The lack of significant differences in internal load suggests that despite varying movement demands, players across positions experience similar physiological strain during training.

### 5.2 Load characteristics of collegiate football players

Match total distance (9,847 ± 1,356 m) was lower than professional players (10,000–13,000 m) reported by Akenhead and Nassis (2016), reflecting the expected performance gap between collegiate and professional populations. The match-to-training load ratio of approximately 1.7 is higher than the 1.3–1.5 ratio typically observed in professional settings (Ravé et al., 2020), suggesting a larger gap between training intensity and match demands in collegiate players. This finding supports the need for training programs that better simulate match conditions, as emphasized by Gabbett (2016) in the training-injury prevention paradox framework. The influence of academic schedules and match density on load-fatigue responses deserves further consideration. Wellness data showed declines in stress levels approaching examinations and sleep quality declines after weekends, indicating that non-training factors substantially affect recovery in collegiate athletes. The observed fatigue incidence rate of 15.2% should be interpreted within the context of our specific monitoring period, which was deliberately scheduled to avoid major examination weeks. Fatigue incidence was notably higher during high-load training weeks (Weeks 4–6) and on Days 1–2 post-match (28.4% vs. 12.6% on regular training days), suggesting that match density is a critical determinant of fatigue accumulation. These context-dependent patterns highlight the importance of considering both academic and athletic schedules when interpreting load monitoring data in collegiate populations.

### 5.3 Applied value of the fatigue prediction model

The XGBoost fusion model (mean AUC = 0.895) substantially outperformed the dummy classifier baseline (AUC = 0.500), confirming genuine predictive validity beyond chance. This performance is comparable to or exceeds recent studies in professional soccer: Rossi et al. (2022a) reported 87% prediction accuracy for wellness forecasting, and Piłka et al. (2023) achieved AUC of 0.90 for injury risk prediction. Notably, our study extends these findings to collegiate populations, demonstrating that machine learning approaches are equally applicable in non-professional settings with appropriate feature selection. Compared to single-indicator models, the multi-sensor fusion approach improved performance by approximately 30.3% over sRPE-only and 23.6% over ACWR-only models. SHAP analysis provided interpretable explanations for the model’s predictions, revealing that declining Wellness scores, elevated ACWR, and reduced morning HRV collectively signal elevated fatigue risk—findings that align with physiological understanding of training adaptation and recovery. Using the more conservative leave-one-subject-out estimate (AUC = 0.847), improvements were 23.3% and 17.0%, respectively. Key predictive features include Wellness score (18.5%), ACWR (16.2%), and morning HRV (13.8%). While our results showed that fatigue incidence increased to 32.6% when ACWR exceeded 1.3, it is noteworthy that ACWR ranked only third in feature importance (16.2%), behind Wellness score (18.5%) and comparable to morning HRV (13.8%). Collectively, these findings suggest that individualized daily fluctuations in subjective status may offer greater predictive value than ratio-based load metrics, supporting the integration of wellness monitoring as a core component of fatigue prediction systems rather than relying primarily on workload ratios such as ACWR. The prominence of Wellness score as the top predictive feature (18.5%) aligns with Simonelli et al. (2025a), who found that subjective wellness indicators—particularly stress and mood—were more influential predictors of match-day fatigue than training load metrics alone. Similarly, Saw et al. (2016) demonstrated that subjective self-report measures often show better sensitivity to training load changes than objective physiological indicators. These converging findings underscore the importance of incorporating daily subjective assessments into comprehensive monitoring frameworks, rather than relying solely on objective workload metrics.

### 5.4 Practical application recommendations

Based on our findings and the international consensus by Bourdon et al. (2017), we propose the following recommendations for collegiate football programs: (1) implement a tiered monitoring strategy with daily HRV/Wellness assessment, session-based GPS/RPE monitoring, and weekly CMJ testing; (2) prioritize daily wellness assessment as a primary early-warning indicator, consistent with Saw et al. (2016); (3) monitor individual load variations relative to each athlete’s baseline rather than applying fixed population thresholds; (4) control weekly load increases within 10% and maintain training monotony below 2.0 (Foster, 1998; Gabbett, 2016). Fatigue warning signals requiring intervention include: HRV decrease >0.5 for 3 consecutive days, CMJ decrease >10%, Wellness score <15, and RPE ≥8 for 2 consecutive days.

### 5.5 Study limitations

Limitations include: single/limited university recruitment; 48-participant sample size; 12-week period not covering complete season; lack of biochemical gold standard validation; specific brand equipment; inadequate control of academic pressure and sleep quality confounders.

### 5.6 Future research directions

Future directions include: multi-center large-sample validation; full-season tracking; exploration of academic pressure-training load interactions; development of mobile real-time monitoring and warning systems.

## 6 Conclusion

Through 12-week multi-sensor monitoring of 48 collegiate football players, this study systematically explored the application effects of wearable devices for training load monitoring and fatigue assessment. Results demonstrate that GPS, heart rate monitors, and subjective questionnaires can effectively collect multidimensional training load data, with significant positive correlations between external and internal load indicators (Player Load and TRIMP: r = 0.81), validating comprehensive monitoring strategy effectiveness. The XGBoost fatigue prediction model achieved AUC of 0.895 (95% CI: 0.862–0.928), with a more conservative leave-one-subject-out estimate of 0.847, both significantly outperforming single-indicator models, with Wellness total score, ACWR, and morning HRV as the most important predictive features. Position-specific load characteristics were observed: midfielders covered greatest total distance, forwards showed highest high-speed running distance, and defenders performed most decelerations. This study fills the research gap in multi-sensor load monitoring for collegiate football players, providing methodological support and practical reference for scientific management of collegiate football training.

## Data Availability

The raw data supporting the conclusions of this article will be made available by the authors, without undue reservation.
